# Composition and metabolic potential of microbial communities in chemically contaminated soils: a multi-dimensional assessment

**DOI:** 10.3389/fmicb.2026.1781030

**Published:** 2026-04-14

**Authors:** Deling Fan, Mengyuan Liang, Lei Wang, Mingqing Liu, Wen Gu, Lili Shi, Zhen Wang, Zheng Fang

**Affiliations:** 1 School of Biological and Pharmaceutical Engineering, Nanjing University of Technology, Nanjing, China; 2 Nanjing Institute of Environmental Science, Ministry of Ecology and Environment, Nanjing, China

**Keywords:** bioremediation, metabolic pathway, network analysis, soil microorganism, soil pollutant

## Abstract

Microbial communities are a crucial component of soil resources and play key roles in various biogeochemical processes. However, in the process of industrialization, the operation, transformation, relocation and other activities of chemical companies inevitably subject soil microorganisms to harsh and complex chemical pressures. Additionally, single-factor biological toxicity tests are difficult to reflect the true situation of contaminated soil environments. A multi-dimensional assessment approach- integrating non-target screening via HPLC-HRMS and 16S rDNA high-throughput sequencing technology with molecular ecological network analysis- was utilized to investigate and compare the soil microbial community structure and composition across seven distinct contaminated sites in China. Additionally, we explored the interactions among microbial species and analyzed the correlations between microorganisms and environmental factors. Phthalates, biphenyls, benzene, polycyclic aromatic hydrocarbons (PAHs), anilines, and phenols were predominant in soil samples, with PAHs serving as the typical representative pollutants across seven contaminated sites. Statistical analysis revealed that *Comamonas aquatica* and Deltaproteobacteria showed significant positive correlations with naphthalene and benzene substituents. Aniline compounds and high-molecular-weight PAHs were primary drivers reducing soil microbial richness and community complexity. Functional prediction based on KEGG and COG indicates that soil microbiomes at different contaminated sites adapt to chemical pollution and remodel community functions through an integrated membrane transport-regulation-catabolism response, characterized by site-specific enrichment of efflux pumps, transport systems, and xenobiotic degradation pathways. This work provides valuable data and scientific support for management of emerging contaminants in chemically contaminated soil.

## 1 Introduction

Soil, though ubiquitous, is an extremely valuable natural resource that performs functions such as agricultural production, ecosystem regulation, and environmental purification (Li et al., 2024; Rillig et al., 2019). Soil microorganisms are an important component of soil, contributing to soil’s rich functionality (Li et al., 2024; Naylor et al., 2022). However, in the process of operation, chemical enterprises inevitably put soil microorganisms under increasing pressure. Specifically, the storage, extraction, and processing of chemical raw materials, as well as the demolition or renovation of plants, aggravate the risk of chemical leakage (Abbaspour et al., 2020; Silva et al., 2023; Hou et al., 2020). These leaked chemical pollutants, including polycyclic aromatic hydrocarbons (PAHs), benzene homologues, organophosphate esters, expose soil microorganisms to unpredictable damage (Kariyawasam et al., 2022; Rada et al., 2019; Yang et al., 2020).

Compared to classical ecotoxicology, chemical pollutants in soil environments occur as mixtures with synergistic or antagonistic interactions, thereby increasing the complexity and scope of research (Piggott et al., 2015; Ramakrishnan et al., 2011; Turschwell et al., 2022). Furthermore, factors such as climate, pH, region, and soil nutrients have a significant impact on the microbial communities’ structure (Deltedesco et al., 2020; Li et al., 2021). To more comprehensively establish the links between the composition and function of microbial communities in contaminated soils, a key strategy is to integrate innovative and traditional techniques with analytical methods (Hellal et al., 2023). Consequently, advanced analytical frameworks capable of processing multi-dimensional datasets are essential to accurately decode the microbial responses in such complex chemical environments (Overy et al., 2021).

The development of high-throughput sequencing techniques has accelerated the analysis, understanding, and utilization of microbial communities (Fierer et al., 2012; Li et al., 2017). To date, significant progress has been made in multiple interdisciplinary fields involving microbial communities, such as agricultural systems, extreme environments, and the impacts of climate change (Gao et al., 2022; Gómez et al., 2016; Zhou et al., 2016; Zhou et al., 2022). However, current research on the composition of microbial communities and interactions between species in soil contaminated by different chemical industries- including coking plants, spice factories, and pesticide factories- remains limited. Soils impacted by chemical enterprises harbor complex pollutants characterized by diverse chemical species, elevated concentrations, and synergistic effects (Piggott et al., 2015; Zuñiga et al., 2017). These pollutants not only exhibit biological toxicity but also present significant technical challenges to the data collection and analysis processes. Therefore, from the perspective of ecological restoration, elucidating the interactions between complex pollutants and microbial communities holds important significant value (Smith et al., 2024; Zuñiga et al., 2017).

In previous studies, the TRIAD and VIKOR methods were combined to effectively assess the ecological risks of different industrial soils in China (Yang et al., 2024). However, the impact of environmental pollutants on microbial populations has not been effectively analyzed. To address this gap, following soil characterization and identification of chemical pollutants, 16S rDNA Illumina high-throughput sequencing was performed (Hiergeist et al., 2016). Then, a multi-dimensional assessment framework integrated with bioinformatic modeling was employed to characterize microbial community structures and predict their functional potential in complex chemically polluted environments. The analysis aimed to identify the types of pollutants that have a significant influence on the structure of microbial communities, as well as the functional differences of microorganisms in complex polluted environments (Bhat et al., 2022; Li et al., 2018).

To systematically comprehend the pollution levels, distribution characteristics, and microbial community structures of seven soil samples from four different industries, a multi-dimensional assessment approach was utilized. This approach involved non-target screening using high-performance liquid chromatography-high resolution mass spectrometry (HPLC-HRMS) and 16S rDNA Illumina high-throughput sequencing, along with molecular ecological network analysis. This work provides critical insights and scientific support for the management of emerging contaminants in chemically contaminated soils. This work provides valuable data and scientific support for the management of emerging contaminants in chemically contaminated soils.

## 2 Materials and methods

### 2.1 Collection and preservation of samples

Soil samples from seven polluted sites in six Chinese cities were collected (Table 1). The description of pollutant profiles is provided in Supplementary Figure S1. Samples from the polluted sites were collected and transported in coolers at 4 °C to minimize the volatilization of organic compounds during transit and storage. Upon arrival at the laboratory, the samples were processed for chemical and microbial analyses.

**Table 1:** Soil samples information.

Site	City	Industry	Longitude	Latitude
HL	Guangzhou	Pigment	113°32′26″	23°05′24″
SG	Shaoguan	Smelter	113°34′21″	24°43′15″
KS	Kunshan City	Spice	120°53′58″	31°16′38″
TY1	Taiyuan	Coking	112°28′53″	37°50′28″
TY2	Taiyuan	Coking	112°28′54″	37°50′32″
XY	Xiaoyi	Coking	111°49′56″	37°05′23″
YQ	Yangquan	Coking	111°20′38″	38°00′36″

### 2.2 Sequencing

#### 2.2.1 Extraction of genome DNA

Total genome DNA was extracted from sample (HL, SG, KS, TY1, TY2, XY, YQ) using the CTAB/SDS method. DNA concentration and purity were monitored on 1% agarose gel electrophoresis. Subsequently, the DNA was diluted to 1 ng/μL using sterile water to standardize the template concentration for downstream analysis.

#### 2.2.2 Amplicon generation

The 16S V3–V4 (341F-806R), 18S V9 (1380F-1510R), and ITS1 (ITS1F-ITS2R) were amplified using the specific primers with the barcode. All PCR reactions were performed in 30 μL reactions with 15 μL of Phusion^®^ High-Fidelity PCR Master Mix (New England Biolabs), 0.2 μM of forward and reverse primers, and about 10 ng template DNA. Thermal cycling consisted of an initial denaturation at 98 °C for 1 min, followed by 30 cycles of denaturation at 98 °C for 10 s, annealing at 50 °C for 30 s, and extension at 72 °C for 60 s, with a final extension at 72 °C for 5 min.

#### 2.2.3 PCR products quantification and qualification

PCR products were mixed with an equal volume of 1X loading buffer (containing SYBR Green) and electrophoresis was performed on 2% agarose gel for detection. Samples exhibiting distinct bands between 400 and 450 bp were selected for further experiments.

#### 2.2.4 PCR products mixing and purification

PCR products were mixed at equal amounts ratios. Then, the pooled mixture was purified using the AxyPrep DNA Gel Extraction Kit (AXYGEN).

#### 2.2.5 Library preparation and sequencing

Sequencing libraries were generated using the NEB Next^®^Ultra™DNA Library Prep Kit for Illumina (NEB, United States) following the manufacturer’s recommendations and index codes were added to attribute sequences to each sample. The library quality was assessed on the Qubit^®^ 2.0 Fluorometer (Thermo Fisher Scientific, United States) and Agilent Bioanalyzer 2,100 system (Agilent Technologies, United States). Finally, the library was sequenced on the Illumina HiSeq 2,500 platform and 250 bp paired-end reads were generated. The raw sequence data generated in this study have been deposited in the NCBI Sequence Read Archive (SRA) and are accessible under the BioProject accession number PRJNA1397987.^1^

### 2.3 Non-target screening of pollutants

#### 2.3.1 Gas chromatography–mass spectrometry for non-target screening

Chemical analysis was performed using an Agilent gas chromatograph equipped with a 5977B mass selective detector. Separation was achieved on a DB-5MS quartz capillary column (30 m × 0.32 mm × 0.25 μm). The injection port temperature was maintained at 280 °C, and a 1 μL sample was injected using a splitless mode with a purge flow of 50 mL/min. The GC oven temperature program started at 80 °C (held for 1 min), increased to 150 °C at 20 °C/min (held for 1 min), and finally reached 300 °C at 10 °C/min (held for 1 min). Helium was used as the carrier gas at a constant flow rate of 1.2 mL/min. The mass spectrometry was operated in EI mode with a full scan range. The ion source and quadrupole temperatures were set to 230 °C and 150 °C, respectively, while the GC–MS interface was kept at 280 °C. Data acquisition and qualitative analysis were conducted using the Agilent MassHunter Workstation software. Initially, the raw data were preprocessed using a deconvolution algorithm, which encompassed peak extraction, alignment, and filtering to derive mass spectra, relative retention times, and predicted molecular formulas. Based on the identified molecular formulas and structural information, screening and matching were performed against various spectral and chemical databases to identify the corresponding compounds.

#### 2.3.2 Ultra-high performance liquid chromatography-quadrupole-orbitrap mass spectrometry for non-targeted screening

The LC separation of chemicals was performed on a Waters ACQUITY UPLC^®^ BEH C18 column (2.1 × 100 mm, 1.7 μm). Non-target screening was conducted in both positive and negative electrospray ionization (ESI) modes using a data-dependent acquisition (DDA) strategy on a UPLC-Q Exactive-Orbitrap-MS. The mass range was set to 100–1,500 *m/z* in Full MS mode. When a precursor ion was detected during the Full MS scan with a response intensity exceeding the predefined threshold (10^4^), the DD-MS2 mode was triggered to acquire corresponding fragment ion information. Target screening was conducted using TraceFinder 4.1 software, which matched the raw UPLC-Q Exactive-Orbitrap-MS data against a combination of commercial spectral libraries (including veterinary drug, pesticide, and food databases provided by Thermo Fisher Scientific) and an in-house library containing 350 Contaminants of Emerging Concern (CECs) developed by our research team. The following parameters were then used for screening: accurate mass error <5 ppm, fragment ion mass error <5 ppm, number of matching fragments ≥2, isotope distribution error <30%, and peak intensity in the sample 5 times higher than in blank soil. After that, the commercial software Compound Discoverer 3.3 was used in combination with mzCloud, ChemSpider, and Mass List databases for screening. Get the preliminary list and filter it after mzCloud Score >70. The identification step for MS results has four different confidence levels. Level a means that the compound is identified and confirmed by reference standards whose accurate mass, retention time, fragmentation, and isotope distribution match. Level b refers to compounds identified by mass spectrometry rather than reference standards, which are considered predicted structures and require further confirmation. Level c and level d mean insufficient evidence exists to propose possible structures.

### 2.4 Data analysis

#### 2.4.1 OTU clustering and species annotation

Sequences analysis was performed using the UPARSE software package using the UPARSE-OTU and UPARSE-OTUref algorithms. In-house Perl scripts were used to analyze alpha (within samples) and beta (among samples) diversity. Sequences with ≥ 97% similarity were assigned to the same OTUs. A representative sequence was picked for each OTU and use the RDP was used to annotate taxonomic information for each representative sequence. To assess alpha diversity, the OTU table was rarefied to calculate three metrics: Chao1 to estimate species richness, Observed Species to determine the number of unique OTUs, and the Shannon index to evaluate community diversity. Rarefaction curves were generated based on these three metrics.

#### 2.4.2 Phylogenic distance and community distribution

The relative abundance and taxonomic distribution of the microbial community, from phylum to species levels, were visualized using the Krona chart. Cluster analysis was preceded by principal component analysis (PCA), which was applied to reduce the dimensionality of the original variables using the QIIME software package. QIIME calculates both weighted and unweighted UniFrac distance, which are phylogenetic measures of beta diversity. Principal Coordinate Analysis (PCoA) and Unweighted Pair Group Method with Arithmetic Mean (UPGMA) clustering were performed based on unweighted UniFrac distances. PCoA was employed for dimensionality reduction to visualize the phylogenetic similarities and clustering patterns among complex, multidimensional microbial datasets. It performs a transformation from a distance matrix to a new set of orthogonal axes, where the maximum variation factor is demonstrated by first principal coordinate, and the second maximum one by the second principal coordinate, and so on. UPGMA was employed as a hierarchical clustering method with average linkage to visualize the similarity patterns derived from the distance matrix.

#### 2.4.3 Statistical analysis

To confirm differences in the abundances of individual taxa between the two groups, STAMP software was utilized. LefSe was used for the quantitative analysis of biomarkers within different groups. This method was designed to analyze data in which the number of species is much higher than the number of samples and to provide biological class explanations to establish statistical significance, biological consistency, and effect-size estimation of predicted biomarkers. To identify differences in microbial communities between the two groups, ANOSIM and ADONIS were performed based on the Bray–Curtis dissimilarity distance matrices.

## 3 Results and discussion

### 3.1 Characterization of soil contaminants

**Figure 1:**
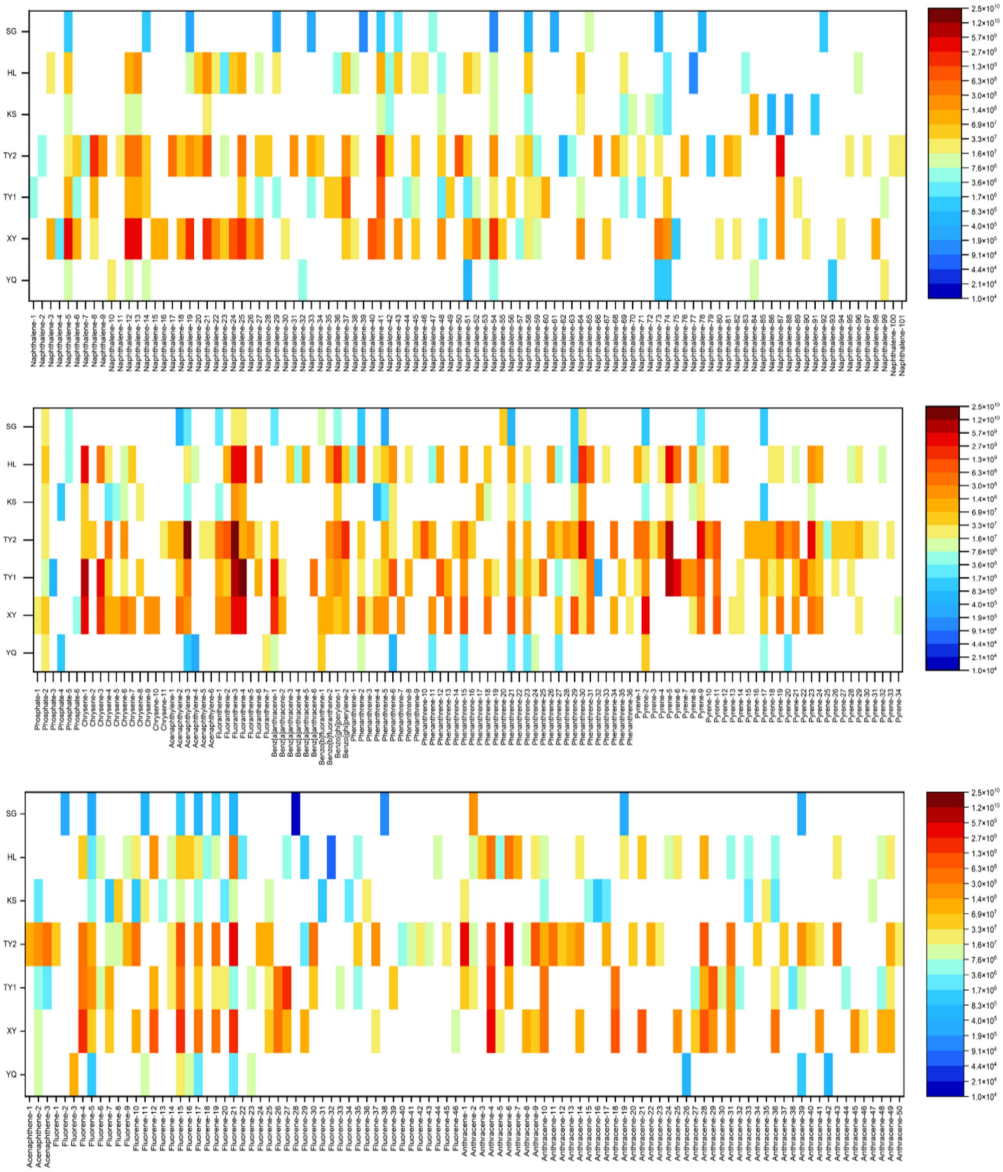
Heatmap of chemical relative abundance in seven polluted soils.

The heterogeneity in soil chemical composition and physical structure affects the microbial community structure (Liu et al., 2018; Philippot et al., 2024). Therefore, the contaminated soil sample collection locations involved seven sites with different climatic conditions to provide more comprehensive environmental information, and their detailed pollution characteristics are shown in Figure 1, Supplementary Table S1, and Supplementary Figure S1. The peak areas in spectral analysis were used as semi-quantitative indicators to compare the relative abundance of pollutants across different samples (Pereira et al., 2021). Non-targeted screening results of soil samples from different contaminated sites by GC-MS and HPLC-HRMS are shown in Supplementary Table S2. Phthalates, biphenyls, benzene, PAHs, anilines, and phenols were predominant in soil samples, as shown in Supplementary Figure S1.

Analysis of compound abundance heatmaps (Figure 1) reveals that the dominant pollutants across soil samples include naphthalene-, fluorene-, anthracene-, phenanthrene-, and pyrene-based compounds. These compounds are typical representatives of the PAH group, which are primarily derived from incomplete combustion of organic matter, industrial discharges, and biomass and fossil fuels (Deng et al., 2024). The widespread presence and relatively high intensities of these compounds suggest that PAH contamination constitutes a major environmental burden in the study area, with potential ecological implications for microbial community structure, soil health, and biogeochemical processes. SG, KS, and YQ exhibited relatively low levels of chemical pollution across all three contaminant categories, and may serve as representative sites for background or baseline environmental conditions. In contrast, TY1, TY2, and XY showed markedly higher levels of contamination, with a broader range of detected compounds and higher peak intensities, indicating complex and diverse pollution profiles. Notably, although both TY1 and TY2 belong to the high-pollution group, their contaminant compositions of PAH differ substantially. Specific pollutants detected, including 1-Methylanthracene, Fluoranthene, Benzo[*k*]fluoranthene, Chrysene, Coronene, likely originated from coal tar and petroleum fractions, reflecting site-specific anthropogenic inputs or industrial activities (Li et al., 2025). The XY point was characterized by alkylated PAHs (such as various methyl naphthalene and dimethyl naphthalene). Based on this chemical characterization of pollutant types and distributions, we further explored how these varying contamination profiles might influence the underlying microbial communities and their associated functions. Given that different organic compounds may impose distinct selective pressures on soil microbiota, the subsequent analysis focuses on microbial taxonomic composition, functional gene profiles, and potential ecological responses under different pollution conditions.

### 3.2 Microbial diversity analysis in different chemically polluted soils

In order to better demonstrate the differences in microbial communities in chemically contaminated soil, the data were analyzed using a combination of multiple analysis indicators. The Alpha diversity analysis of samples was measured using observed species and Shannon index, respectively. Quality control data statistical analysis was shown in Supplementary Table S3. The results of rarefaction curve (Supplementary Figure S2a) showed that the number of species tends to stabilize when the number of sequences reached about 40,000. Furthermore, species richness varied significantly among different contaminated soil samples. As shown in Supplementary Figure S2b, the Shannon index of each sample rose rapidly as the sequence number increased, and leveled off after reaching a certain sequence number. It indicated that the sequencing depth was sufficient to represent the diversity of different samples (Hill et al., 2003). The distribution and differences in species richness between different contaminated land sample groups were compared, the results revealed that the median species richness was lowest in the KS group, while the XY group exhibited the highest median species richness. Combined with Supplementary Figure S1, it showed that the concentration of aniline compounds has a negative correlation with the abundance of microorganisms in contaminated soil. This effect was also consistent with the bactericidal properties of aniline compounds (Huang et al., 2018; Mohammed et al., 2020). The US Environmental Protection Agency (EPA) announced a proposal to designate aniline as high priority substances for risk assessment under Section 6 (b) of the Toxic Substances Control Act (TSCA), on July 24, 2024. Meanwhile, the TY2 group showed the longest whiskers and exhibited significant variability in observed species count. TY2 soil sample had relatively low species richness, which may be attributed to the intense selective pressure of site-specific contaminants. 7-Methylquinoline and 3,4-dimethylpyridine were identified as the distinctive markers that differentiated these samples from the others. Naphtho[1,2-*b*]furan, Anthracene, Benzo[*a*]pyrene, and 2-Nitrophenol were higher than other samples. Combined with Supplementary Figure S2a, it showed that this situation was caused by the deviation of the TY2-3 parallel group from TY2-1 and TY2-2. The other groups (HL, SG, TY1, XY, YQ) had more similar medians and interquartile ranges in terms of observed species, and their data distributions were more concentrated, indicating smaller differences among the samples within these groups.

The beta diversity analysis of intergroup differences based on weighted Unifrac distance illustrated the differences in microbial community structure among the different groups (Fukuyama et al., 2012). The weighted Unifrac distances for the KS and YQ groups were relatively large, indicating that their microbial community structures differed significantly from those of the other groups. In contrast, the distances for the HL, TY1 and TY2 groups were relatively small, suggesting that their microbial community structures were more similar to those of the other groups. The Total Organic Carbon (TOC) in the KS and YQ groups was relatively low, and limited nutrients tend to intensify the competitive relationship among microbial populations (Hibbing et al., 2010).

### 3.3 Composition of microbial communities’ structure in different chemically polluted soils

**Figure 2:**
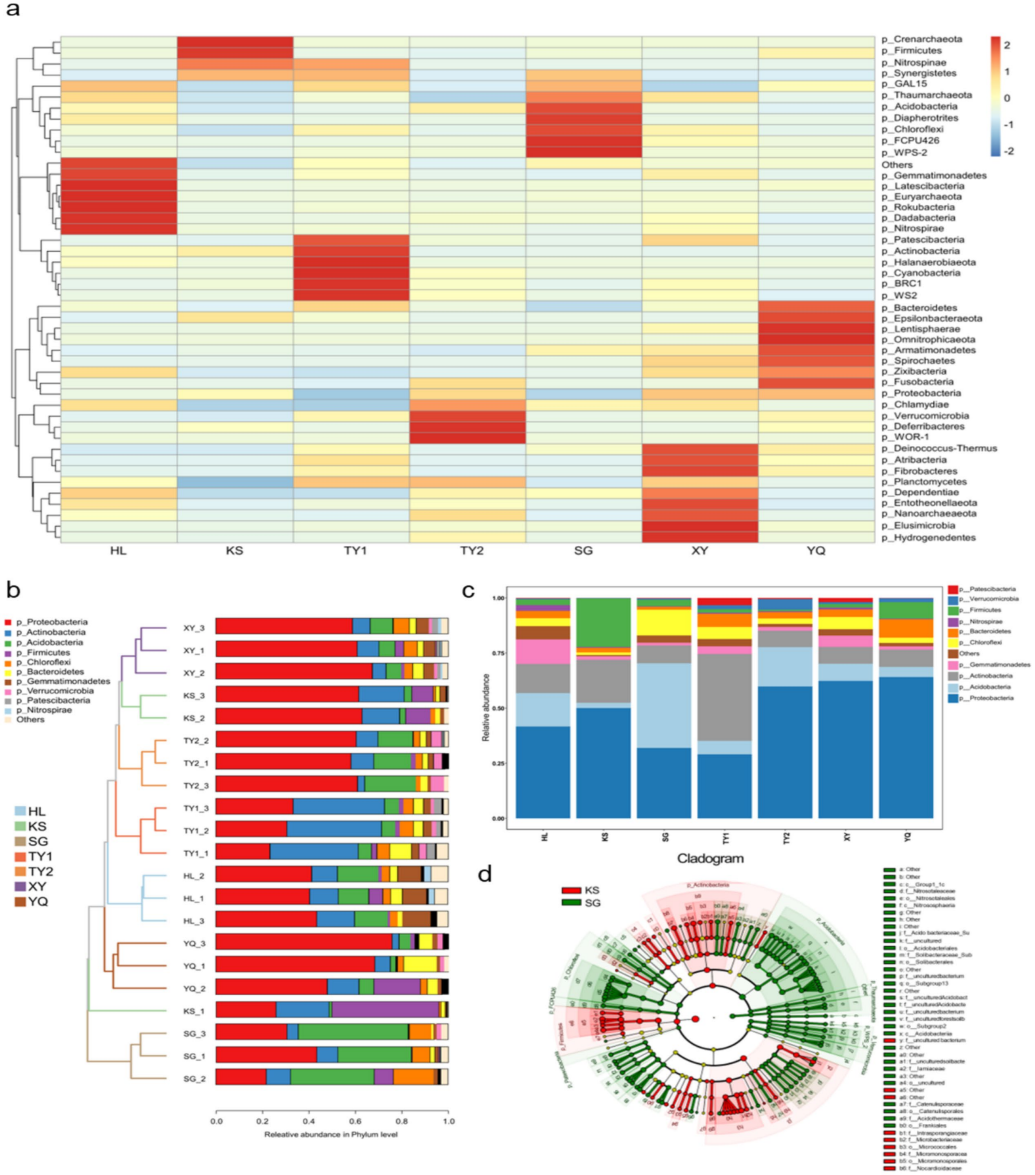
Comparative analysis of microbial communities’ structures in different contaminated soils. **(a)** Community heatmap of microbial communities’ structure; **(b)** UPGMA clustering tree based on weighted Unifrac distance and stacked bar chart of community structure; **(c)** stacked bar chart of relative abundance at phylum level; **(d)** cladogram of sample groups of KS and SG contaminated soils.

Based on heatmap analysis, the relative abundance of each phylum in different contaminated soil groups was displayed in Figure 2a. The KS group showed a relatively high abundance of multiple phyla, such as Crenarchaeota, Firmicutes, and Nitrospinae. The common characteristic of these bacteria is their robust environmental adaptability (Bandopadhyay and Shade, 2024). A variety of bacteria with strong environmental adaptability were found in highly abundant phyla in the YQ group, such as Epsilonbacteraeota, Omnitrophicaeota, Armatimonadetes, and Zixibacteria. The relatively high abundance of Crenarchaeota (ability to convert sulfide), Nitrospinaeand (ability to oxidize nitrates), Epsilonbacteraeota (ability to oxidize sulfides) also proved that in a limited organic carbon environment (Waite et al., 2017), bacteria with the ability to decompose and utilize pollutants were more competitive. Combined with Supplementary Figure S1, the lower pollution level put the SG group in relatively stable environments, leading to a more balanced relative abundance.

In the field of environmental biology, UPGMA is a commonly used clustering analysis method (Loewenstein et al., 2008). To explore the similarities among different sample groups from various polluted sites, UPGMA clustering analysis was performed on the samples to construct a clustering tree. UPGMA clustering analysis was conducted based on the Weighted Unifrac and Unweighted Unifrac distance matrices. The clustering results were integrated with the stacked bar chart of community structures at the bacterial phylum level for each sample, as shown in Figure 2b. The results showed that Proteobacteria dominated in multiple groups (such as KS, TY2, YQ, XY), accounting for approximately 50% of the microbial community. The results also indicated that the SG, TY1, and HL groups were distinct from the KS, TY2, YQ, and XY sample groups. Specifically, in the SG and TY1 group, Actinobacteria constituted around 40% of the community. In the HL group, Proteobacteria was dominant, making up approximately 40%. Notably, in the KS group (KS-1), Firmicutes was the most dominant, reaching up to 48% of the community, while in KS-2 and KS-3, it was around 10%. The relative abundance of Proteobacteria in KS-2 and KS-3 samples reached about 50%, which was similar to the situation of KS, TY2, YQ, XY groups. The reason for the large differences in the parallel samples of the KS group may be that the contaminated soil was affected by pollutants from spice and pesticide factories at the same time. This dual effect was non-uniform, which also indicates the necessity of parallel sampling in soil microbial analysis.

As shown in Figure 2c, the stacked bar chart quantifies the relative abundance of each phylum across the different contaminated soil samples. Proteobacteria was the most abundant microbial phylum in all samples. The community composition of KS and SG samples showed higher diversity, which may be related to the types of pollutants and environmental conditions in which they are located. We speculated that the diversity of the community composition of the KS sample group was related to the heterogeneity and complexity of pollutants in the contaminated soil, while the diversity of the community composition of the SG sample group was related to the low level of pollutants. Therefore, KS and SG samples deserve further comparative analysis to obtain a more detailed relationship between soil contaminants and microbial populations.

The LEfSe analysis uses linear discriminant analysis (LDA) based on taxonomic composition to identify communities or species that significantly influence the differentiation of samples according to different grouping conditions. As shown in Figure 2d, based on the phylogenetic tree (cladogram), the microbial community composition of the two sample groups (KS: Red color and SG: Green color) at different taxonomic levels was displayed. Specifically, some families and genera in Actinobacteria (such as Microbacteriaceae, *Micrococcus*) were significantly enriched in the KS group; while some families and genera in Acidobacteria (such as Solibacteraceae*, Solibacter*) were significantly enriched in the SG group. Based on PCA (Supplementary Figure S3), the significant difference in microbial community composition between the KS group and the SG group was further confirmed.

**Figure 3:**
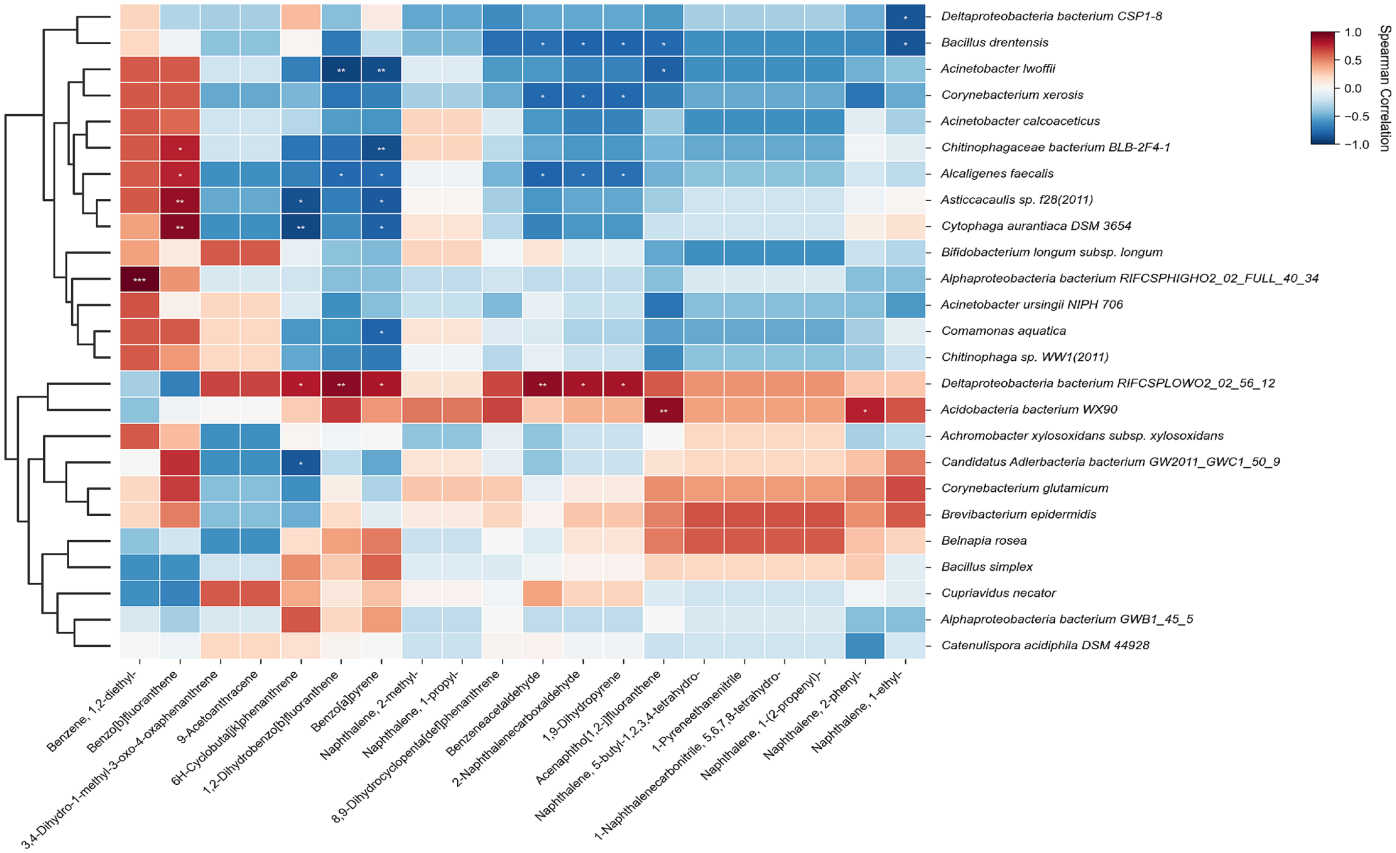
Correlation between key pollutants and microbial taxa.

The statistical analysis revealed that several aromatic pollutants—specifically benzene and naphthalene derivatives, tetrachlorobenzene, and PAH residues—exhibited significant correlations with key microbial species (marked with asterisks for *p* < 0.05, 0.01, or 0.001) (Figure 3). Notably, *Comamonas aquatica* and Deltaproteobacteria showed significant positive correlations with naphthalene and benzene substituents. This robust association points toward the possibility that these taxa are potentially involved in the degradation of these aromatic compounds or exhibit high physiological tolerance to their toxicity (Yuan et al., 2020; Son et al., 2025). In contrast, *Acinetobacter baumannii*, Alphaproteobacteria and Deltaproteobacteria showed significant negative correlations with aniline compounds and high-molecular-weight PAHs. This pattern indicates that these microbes were either directly inhibited by the chemical stress or outcompeted within their ecological niches by more resilient pollutant-degrading populations. These findings underscore the selective pressure exerted by industrial contaminants in shaping the functional structure of the soil microbiome. Son et al. (2025) reported that the bacterium *Pseudomonas* sp. MC1 harbors an 81-kb metabolic plasmid encoding enzymes involved in the conversion of naphthalene to salicylate. In this pathway, the nahX gene within the lower naphthalene degradation operon was found to encode a 40-amino acid protein critical for the catabolic process. Similarly, Yuan et al. (2020) investigated the isolated strain *Comamonas* sp. ZF-3 and found that it exhibited high biodegradability toward phenolic and heterocyclic compounds in complex industrial wastewater. The results showed that accumulation of pollutants could caused severe oxidative stress, inhibit the growth of plants, and disrupted normal microbial metabolic processes, while increasing the relative abundance of PAH degrading bacteria (*Sphingobacterium*, *Pseudomonas*, *Rhodococcus* and *Massilia* etc.) (Song et al., 2023; Zhou et al., 2025).

### 3.4 Prediction of microbial community functions in different contaminated soils

**Figure 4:**
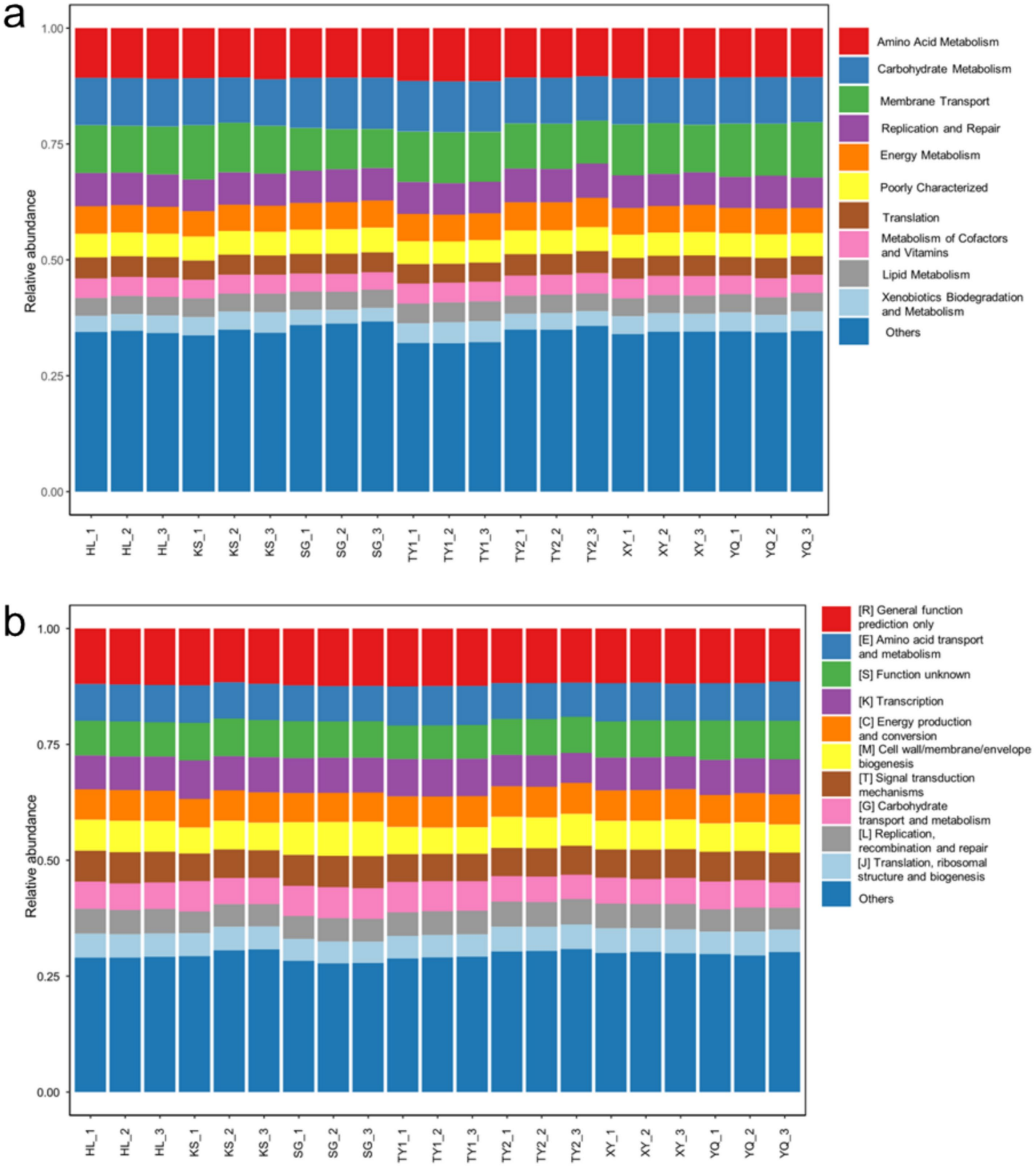
Bar chart of relative abundances for KEGG **(a)** and COG **(b)** functional categories.

Kyoto Encyclopedia of Genes and Genomes (KEGG) and Clusters of Orthologous Groups (COG) are two commonly used functional annotation and classification systems, respectively providing different biological information and functional analysis methods (Galperin et al., 2020; Kanehisa and Goto, 2000). Combined KEGG and COG analysis provide a more comprehensive and in-depth analysis of the functional characteristics of microbial communities in contaminated soil. As shown in Figure 4a, the functional composition of different samples under the KEGG classification system, where the bars of each color represent different functional categories. The main functional categories with the highest proportion include amino acid metabolism, carbohydrate metabolism, and membrane transport. Among the above functional categories, the membrane transport function showed greater differences in samples from different contaminated soils. The adaptability of microbial cell membranes largely determines the survival of cells (Hąc-Wydro et al., 2019). This result also highlights that microorganisms form a selective barrier between the inside of the cell and the outside world (Murínová and Dercová, 2014). In addition, the relative abundance and composition of the “other” region were different. This difference may be due to different factors such as environmental conditions, nutrient sources, microbial interactions, etc., resulting in unique functional composition. Similarly, the results based on the COG classification system (Figure 4b) were also consistent with the KEGG system, mainly: general function prediction only, Amino acid transport and metabolism, function unknown, etc. The homogeneous results obtained by both analysis modes indicated that the microbial communities were relatively stable in these contaminated soils. Therefore, by monitoring changes in these functional categories, environmental quality and the health of microbial communities can be assessed.

**Figure 5:**
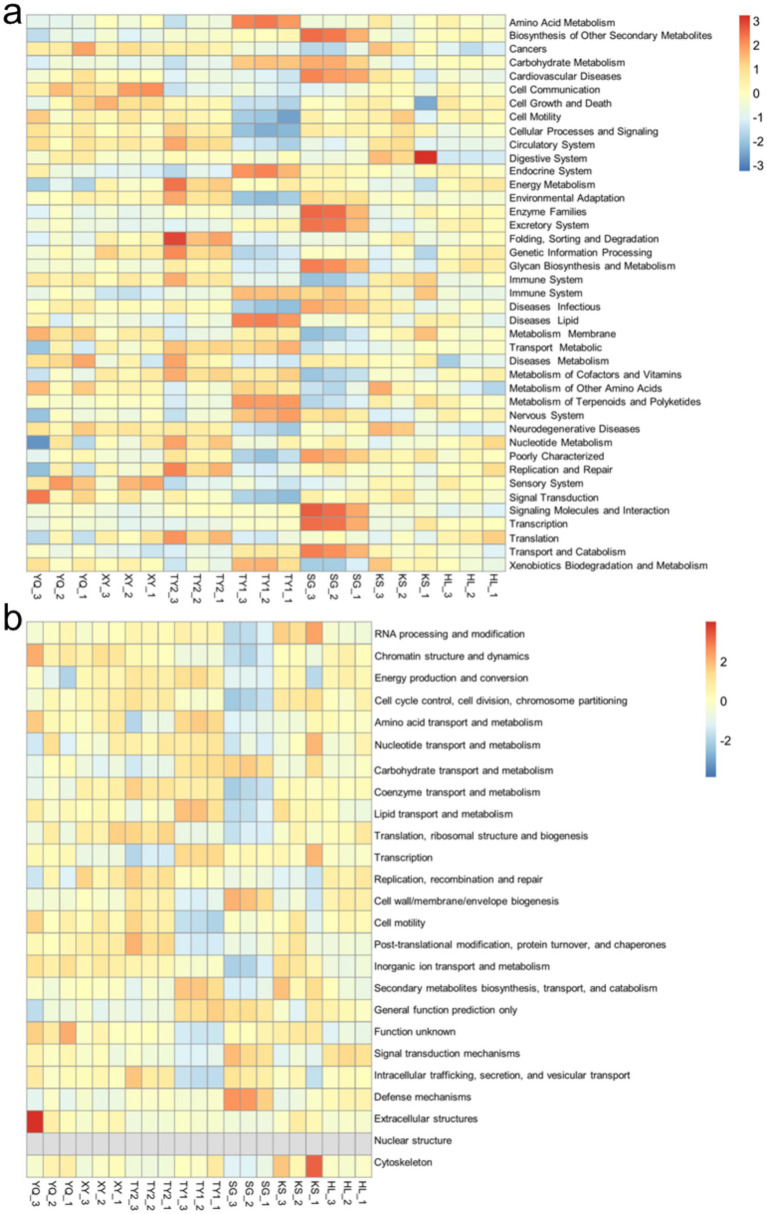
Heatmap analysis of KEGG **(a)** and COG **(b)** functional predictions.

The relative abundance data were then normalized and KEGG and COG heatmaps were generated (Figure 5) to visually display the changes in the functions and metabolic pathways of microbial communities in different samples. Specifically, in Figure 5a, the TY1 sample group showed high abundance in multiple areas such as amino acid metabolism, endocrine system, lipid metabolism, indicating that the microbial communities in the TY1 sample group could utilize a variety of different carbon and energy sources to sustain growth and reproduction. The SG sample group showed high abundance in multiple areas such as signaling molecules and interaction, transcription, enzyme families, excretory system, transport and catabolism, etc., indicating that the microbial communities in the SG sample group may possess stronger adaptability and degradation capabilities for specific chemical pollutants. The relative abundance of each function of the microbial community in HL and KY sample groups was mainly concentrated in the range of 0–1. This suggested that the ecosystem in the HL and KY sample groups was relatively stable, with a well-coordinated microbial community that was better adapted to chemical pollutants. The uniform distribution of the relative abundances of HL and KY sample groups in the COG heatmap (Figure 5b) also proved this point.

The observed enrichment of specific taxa likely reflects distinct adaptive strategies encoded in their genomes. The prevalence of PAHs favored genera such as *Pseudomonas* and *Sphingobacterium*, which have been widely reported to harbor ring-hydroxylating dioxygenase genes (e.g., nidA, nahAc) (Ghosal et al., 2016). Consistent with this taxonomic shift, our functional prediction revealed an increased potential for xenobiotic biodegradation, suggesting that the community possesses the genetic repertoire necessary for aromatic ring cleavage. In contrast, the negative association between aniline and high-molecular-weight PAHs richness points to toxicity mediated filtering. This is supported by the predicted functional profiles, where membrane transport modules showed significant site-specific variations. The enrichment of predicted efflux pump genes (e.g., transporters in the ABC and RND families) in these samples implies a genomic potential for countering membrane toxicity, a common resistance mechanism against solvent-like pollutants (Liu et al., 2021). Although these results are predictive, they highlight an integrated genomic framework-combining efflux capability with catabolic versatility-that likely underpins the community’s persistence under complex chemical stress.

## 4 Conclusion

Microbial communities are an integral component of soil resources and serve as key indicators of soil quality, characteristics, and function. The development of 16 s rDNA technology provides the ability to analyze large quantities of microbial community structure information. Based on this, we combined a variety of analytical methods to reduce the impact of chemical pollutants on the detection and analysis process, and obtained structural information on soil microbial community of different types of pollution. The research results also pointed out that in the face of stress from different pollution sources, the cell membrane-related functionality of microorganisms showed differences. PAHs were typical representative pollutants in soil at seven contaminated sites. *Comamonas aquatica* and Deltaproteobacteria showed significant positive correlations with naphthalene and benzene substituents. In contrast, *Acinetobacter baumannii*, Alphaproteobacteria and Deltaproteobacteria showed significant negative correlations with aniline compounds and high molecular weight PAHs. Our findings show that soil microbial communities maintain resilience through an integrated transport-regulation-catabolism response. This mechanism is characterized by the precise enrichment of diverse membrane transporters and catabolic pathways. These findings highlight compound-specific links between membrane functions and pollutants, informing strategies to strengthen tolerance in pollutant-degrading strains through targeted functional modulation. Collectively, this study provides actionable evidence and scientific support for the management of emerging contaminants in chemically contaminated soils.

## Data Availability

The data presented in the study are deposited in the NCBI SRA repository, accession number PRJNA 1397987 (https://www.ncbi.nlm.nih.gov/bioproject/PRJNA1397987).

## Footnotes

1
https://www.ncbi.nlm.nih.gov/bioproject/PRJNA1397987
