# Supplementary material for: Composition and metabolic potential of microbial communities in chemically contaminated soils: a multi-dimensional assessment
Source: Front Microbiol. 2026 Apr 14;17:1781030. doi: 10.3389/fmicb.2026.1781030 (PMC13121128; doi:10.3389/fmicb.2026.1781030)
Supplement: Supplementary file 1 [file Data_Sheet_1.pdf]

Composition and metabolic potential of microbial communities in chemically contaminated soils: A multi-dimensional assessments

Deling Fan[a,b], Mengyuan Liang[b], Lei Wang[b], Mingqing Liu[b], Wen Gu[b], Lili Shi[b], Zhen Wang[b*], Zheng Fang[a*]

[a]School of Biological and Pharmaceutical Engineering, Nanjing University of Technology, Nanjing, 211816, China

[b]Nanjing Institute of Environmental Science, Ministry of Ecology and Environment, Nanjing, 210042, China

Address for Correspondence:

Zhen Wang*

Nanjing Institute of Environmental Science, Ministry of Ecology and Environment, Nanjing, 210042, China

Email: wangzhen@nies,org

Zheng Fang*

School of Biological and Pharmaceutical Engineering, Nanjing University of Technology

Email: fzcpu@njtech.edu.cn

*Corresponding authors

E-mails:wangzhen@nies.org (Zhen Wang), fzcpu@njtech.edu.cn (Zheng Fang).

Experimental Procedures

Extraction of organic contaminants from soil samples

Weigh 5.0 g of soil sample into a 50ml polytetrafluoroethylene centrifuge tube, then add 3g of anhydrous sodium sulfate and mix well. Physical and chemical properties of contaminated soil leaching solution were detected as the literature[1,2]. Then add 20 mL of dichloromethane and ethyl acetate (volume ratio 1:1) mixed solvent, vortex for 30 s and then sonicate for 20 min. Then centrifuge in a refrigerated centrifuge for 5 minutes at a speed of 6500 r/min. Collect the supernatant through qualitative filter paper and add 20 mL of extraction solvent to the centrifuge tube again. Repeat the above operation twice and combine the supernatants. The extract was concentrated under reduced pressure to 2 mL, and then purified through a silica gel column. The eluent was dichloromethane-ethyl acetate (volume ratio 1:1). The eluate was concentrated to nearly dryness by blowing nitrogen, and the volume of methanol was adjusted to 1 mL, and then analyzed by Gas chromatography–mass spectrometry (GC-MS) and ultra-high performance liquid chromatography-quadrupole-orbitrap mass spectrometry (UPLC-QE-Orbitrap-MS) respectively.

Gas chromatography–mass spectrometry for untargeted screening

Instrument: Agilent gas chromatograph tandem 5977B mass spectrometer; Chromatographic column: DB-5MS (30 m×0.32 mm×0.25 μm) quartz capillary column; Injection port temperature: 280 °C; Injection method: purge flow to the split port 50 mL/min; Programmed temperature rise: 80 °C for 1 min, 20 °C/min to 150 °C for 1 min, then 10 °C/min to 300 °C for 1 min; Carrier gas: helium; Flow rate: 1.2 mL/min; Injection volume: 1 μL.

Mass spectrometry conditions: EI source; Ion source temperature: 230 °C; Quadrupole temperature: 150 °C; Scanning mode: full scan; Solvent delay 3 min; GC-MS interface temperature: 280 °C.

Data Processing and Analysis: Import the chromatograms obtained from GC-MS detection into Agilent MassHunter Workstation software for qualitative analysis. First, use the deconvolution function to process the collected data, which includes peak extraction, alignment, and filtering, to obtain mass spectra, relative retention times, and predicted molecular formulas. Based on the obtained list of molecular formulas and structural

information, perform screening and matching through mass spectrometry databases and chemical databases to obtain a corresponding list of compounds.

Ultra-high performance liquid chromatography-quadrupole-orbitrap mass spectrometry for untargeted screening

The LC separation of chemicals was performed on a Waters ACQUITY UPLC® BEH C18 column (2.1 × 100 mm, 1.7 μm). Non-target screening was conducted in both positive and negative electrospray ionization (ESI) modes and data-dependent second-order daughter ion scanning mode, coupled with UPLC-QE-Orbitrap-MS. The mass range (100-1500 m/z) was set to Full MS mode. When the parent ion in the sequence table is detected by the first-level full scan and the response intensity exceeds the set threshold (precursor intensity threshold of 1e4), the DD-MS2 scanning mode is triggered, and the second-level fragment ion information of the parent ion is obtained.

The original UPLC-QE-Orbitrap-MS data was imported into TraceFinder 4.1 software and combined with the veterinary drugs, pesticides, and food databases established by Thermo Fisher Scientific and the self-built database containing 350 Contaminants of Emerging Concern (CECs) established by the research team for target screening. The following parameters were then used for screening: accurate mass error <5 ppm, fragment ion mass error < 5 ppm, number of matching fragments ≥ 1, isotope distribution $< 30\%$, and peak intensity in the sample 5 times higher than in blank soil. After that, the commercial software Compound Discoverer 3.3 was used in combination with MzCloud, ChemSpider, and Mass List databases for screening. Get the preliminary list and filter it after mzCloud Score > 70. The identification step for MS results has four different confidence levels. Grade A means that the compound is identified and confirmed by reference standards whose accurate mass, retention time, fragmentation, and isotope distribution match. Level b refers to compounds identified by mass spectrometry rather than reference standards, which are considered predicted structures and require further confirmation. Level c and level d means insufficient evidence exists to propose possible structures.

Table S1 Physicochemical properties of the leachates from contaminated soil samples

**Sample**	**Industry**	**pH**[1]	**Electrical conductivity(*uS/cm*)**[2]	**Redox (mV)**[3]	**Organic carbon content**[4]**(TOC,** g/kg)
KS	Spice	7.97	480	156.1	22.64
XY	Coking	7.75	2.48×10^3	103.6	7.55
YQ	Coking	7.97	428	73.4	11.57
HL	Pigment	7.63	178.4	103.9	10.23
SG	Smelter	8.04	125.6	98.7	9.74
TY1	Coking	7.31	104.3	141.5	15.18
TY2	Coking	7.26	218.6	187.6	7.11

[1]**Soil- Deternimation of pH-Potentiometry**

[2]**Soil quality- Determination of conductivity-Electrode method(HJ 802-2016)**

[3]**Soil - Determination of redox potential - Potentiometric method (HJ 746-2015)**

[4]**Soil-Determination of organic carbon-Combustion oxidation nondispersive infrared absorption method (HJ 695-2014).**

Table S2 Non-targeted screening results of soil samples from different contaminated sites (number of pollutants)

Sample	**XY**	**YQ**	**KS**	**HL**	**SG**	**TY1**	**TY2**
Pollutants in preliminary screening	2570	1979	2834	2206	1974	2800	2814
Specific pollutants	1094	746	1370	781	750	1079	1298
Pollutants in qualitative Screening	663	486	637	543	335	715	637
Toxic and harmful pollutants	160	98	165	134	65	155	185

*Summarized after analysis and identification by GC-MS and UPLC-QE-Orbitrap-MS

Table S3 Quality control data statistical analysis table

Sample*	Raw PE (#)	Combined (#)	Qualified (#)	Nochime (#)	Base (nt)	AvgLen (nt)	Q20	Q30	GC (%)	Effective (%)
YQ.1	101,257	91,825	87,543	60,010	25,097,671	418	97.64	92.87	54.19	59.27
YQ.2	102,941	83,238	78,514	60,492	25,176,999	416	97.56	92.81	54.66	58.76
YQ.3	101,023	88,141	82,942	65,831	27,780,719	422	97.54	92.72	54.64	65.16
KS.1	93,953	83,871	79,365	60,087	25,340,863	422	97.38	92.36	56.45	63.95
KS.2	84,238	73,755	69,606	57,125	23,972,795	420	97.51	92.69	55.52	67.81
KS.3	110,399	100,671	95,685	67,277	28,076,075	417	97.53	92.75	55.64	60.94
XY.1	96,443	86,615	82,792	65,657	27,368,678	417	97.6	92.89	55.71	68.08
XY.2	60,438	53,505	50,700	40,916	17,111,059	418	97.52	92.61	55.5	67.7
XY.3	104,144	94,832	90,546	66,206	27,520,921	416	97.61	92.9	55.92	63.57
HL.1	100,807	89,401	85,612	60,231	25,082,737	416	97.67	93.02	57.14	59.75
HL.2	104,770	93,364	87,878	62,185	25,903,070	417	97.53	92.68	57.49	59.35
HL.3	104,834	95,845	91,321	65,045	27,096,119	417	97.68	93.04	57.92	62.05
SG.1	98,276	90,232	86,544	62,835	25,963,599	413	97.78	93.24	56.78	63.94
SG.2	100,471	90,723	86,911	65,985	27,150,237	411	97.79	93.27	57.18	65.68
SG.3	107,192	98,503	94,373	68,455	28,250,561	413	97.78	93.27	57.44	63.86
TY1.1	95,845	86,072	82,078	61,470	25,427,248	414	97.62	92.87	56.43	64.13
TY1.2	112,501	100,469	95,980	67,275	27,796,258	413	97.68	93.05	56.92	59.8
TY1.3	104,222	94,462	89,678	65,228	26,988,830	414	97.56	92.76	56.63	62.59
TY2.1	110,343	101,123	96,403	65,858	27,593,556	419	97.49	92.54	55.57	59.68
TY2.2	68,240	60,637	57,549	46,878	19,590,444	418	97.69	93.07	55.56	68.7
TY2.3	91,425	84,632	80,594	60,364	25,431,578	421	97.58	92.88	55.01	66.03

*Three parallel samples were selected for each sample and tested

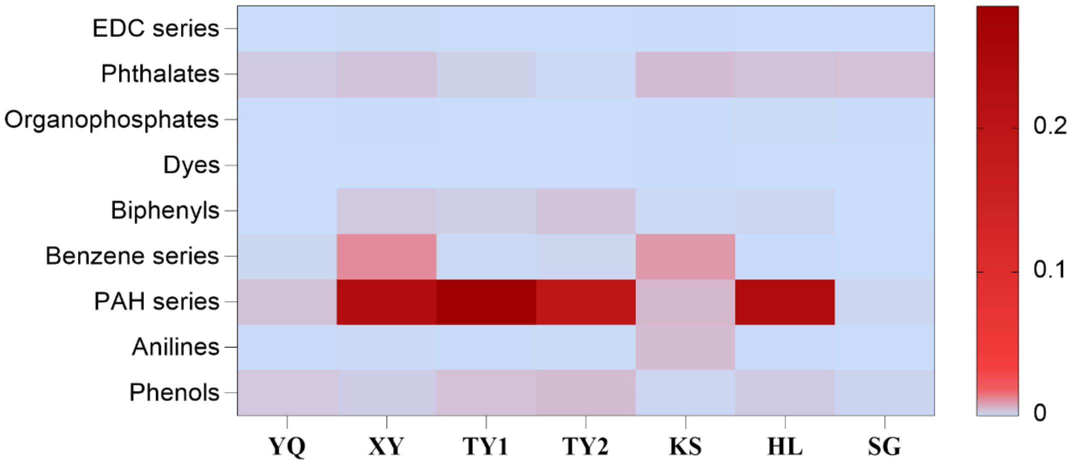

Figure S1 Distribution map of toxic and harmful pollutants in soil at 7 contaminated sites.

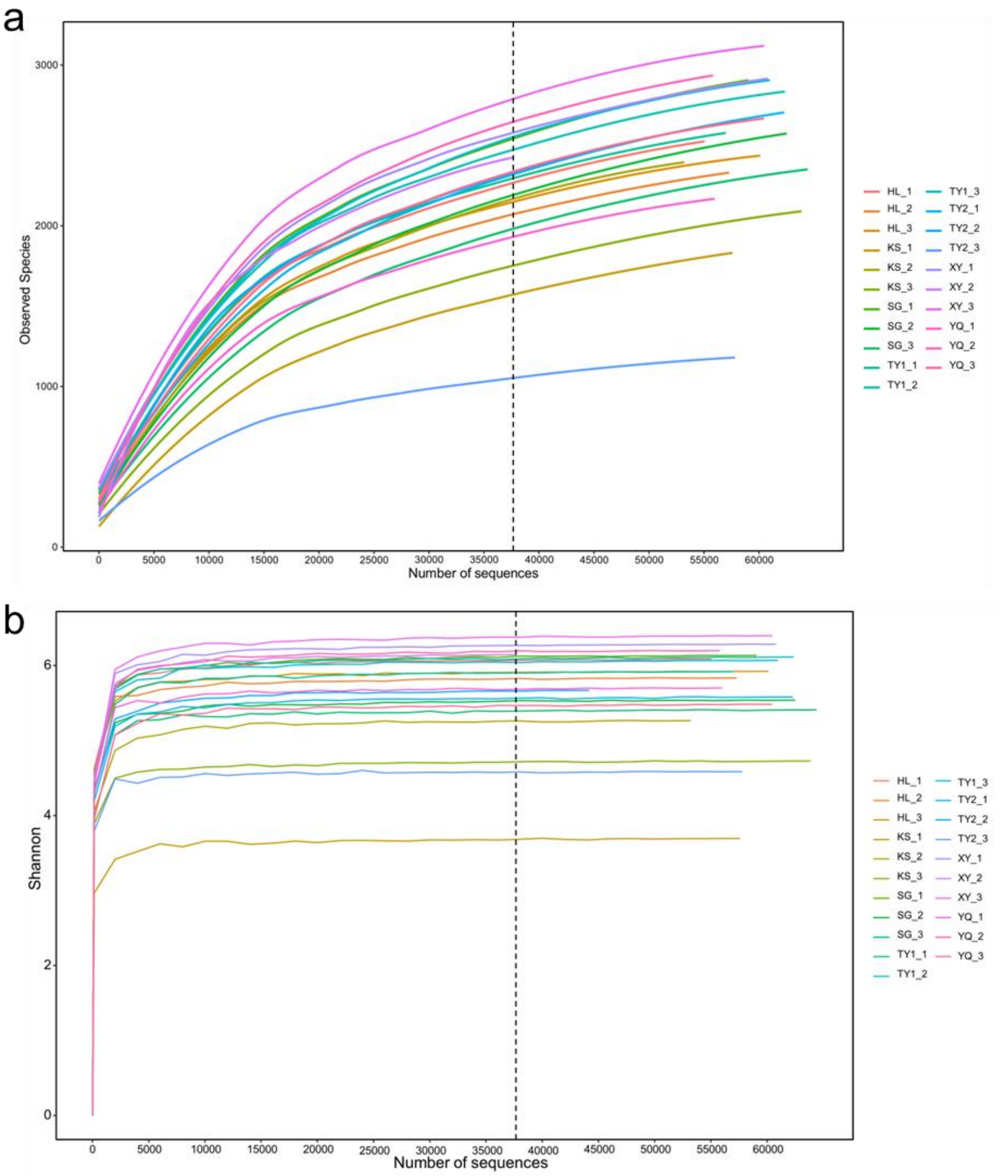

Figure S2 Alpha diversity metrics of microbial communities in different contaminated soils. **a,** Rarefaction curve of different samples; **b,** Shannon curve of different samples. Each contaminated soil sample group was sampled three times in parallel.

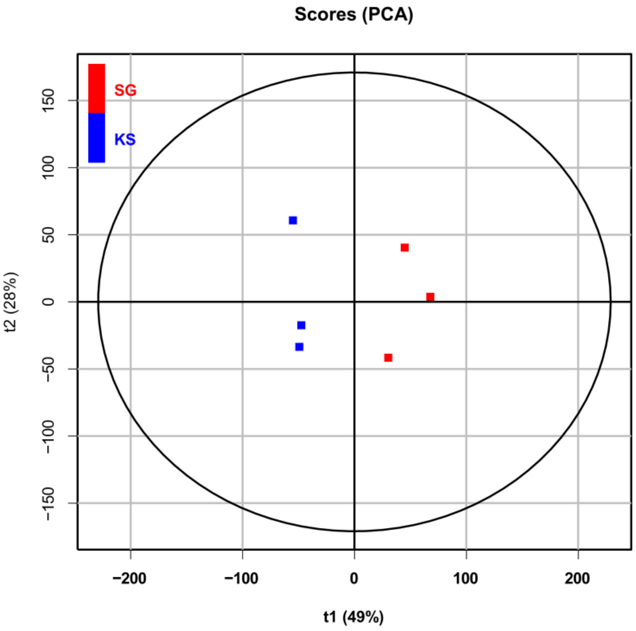

Figure S3 Principal component analysis of KEGG functional categories for SG and KS groups.

Reference

[1] Q. Zhang, et al., Spatial occurrence and composition profile of organophosphate esters (OPEs) in farmland soils from different regions of China: Implications for human exposure, Environmental Pollution, 276 (2021), 116729. https://doi.org/10.1016/j.envpol.2021.116729

[2] J. Rousk, et al., Soil bacterial and fungal communities across a pH gradient in an arable soil, The ISME Journal,2010,4:1340-1351.
